# Supplementary material for: Do Semaphorins Play a Role in Development of Fibrosis in Patients with Nonalcoholic Fatty Liver Disease?
Source: Biomedicines. 2022 Nov 23;10(12):3014. doi: 10.3390/biomedicines10123014 (PMC9775767; doi:10.3390/biomedicines10123014)
Supplement: Supplementary file 1 [file biomedicines-10-03014-s001.zip › biomedicines-2052777-supplementary.pdf]

SUPPLEMENTARY TABLE S1.

Serum concentrations of semaphorins in patients with non-alcoholic fatty liver disease and healthy controls

**NAFLD**	**SEMA3A**	**SEMA3C**	**SEMA4A**	**SEMA4D**	**SEMA5A**	**SEMA7A**
Minimum	9	1,2	3,1	3,3	1,3	0,03
25% Percentile	12	10	4,2	13	7	0,64
Median	16	13	5,5	45	21	1,2
75% Percentile	22	19	6,6	85	44	1,7
Maximum	27	36	32	118	159	3,3
Mean	17	15	7,4	49	30	1,4
Std. Deviation	5,5	8,7	7,2	36	32	0,9
Std. Error of Mean	0,94	2,2	1,9	6,1	5,5	0,15

**HEALTHY CONTROLS**	**SEMA3A**	**SEMA3C**	**SEMA4A**	**SEMA4D**	**SEMA5A**	**SEMA7A**
Minimum	6,9	5,4	3,5	13	0,13	0,03
25% Percentile	16	13	4,7	55	2,9	0,47
Median	22	18	5,6	67	8,5	0,92
75% Percentile	25	32	7,5	84	28	1,3
Maximum	68	99	21	135	72	4,7
Mean	23	26	6,9	70	18	1
Std. Deviation	10	21	3,5	25	20	0,82
Std. Error of Mean	1	2,6	0,44	2,6	2,1	0,084
p-value	0,0002	0,0163	0,469	0,0004	0,009	0,0332
Difference	-6,5	-5,1	-0,14	-22	12	0,25
Difference (Hodges-Lehmann)	-5,4	-5,6	-0,39	-24	6,5	0,29
95% CI of difference	-8,4 to -2,3	-13 to -1,0	-1,5 to 0,69	-36 to -10	1,4 to 14	0,020 to 0,59

SUPPLEMENTARY TABLE S2.
Serum concentrations of semaphorins according to steatosis grade in patients with non-alcoholic fatty liver disease

**STEATOSIS GRADE 3**	**SEMA3A**	**SEMA3C**	**SEMA4A**	**SEMA4D**	**SEMA5A**	**SEMA7A**
Minimum	8	5,4	3,5	31	0,13	0,03
25% Percentile	16	13	4,7	54	2,9	0,47
Median	22	20	5,8	67	8,2	0,87
75% Percentile	25	35	8,5	87	21	1,3
Maximum	68	99	17	135	57	4,7
Mean	22	28	7,2	70	15	0,99
Std. Deviation	9,4	23	3,5	25	18	0,78
Std. Error of Mean	1,2	3,6	0,55	3,2	2,3	0,1

**STEATOSIS GRADE 2**	**SEMA3A**	**SEMA3C**	**SEMA4A**	**SEMA4D**	**SEMA5A**	**SEMA7A**
Minimum	7,8	8,5	3,5	29	0,37	0,03
25% Percentile	19	14	4,4	48	2	0,5
Median	22	18	5,1	67	6,1	0,83
75% Percentile	28	42	5,9	75	33	1,1
Maximum	51	78	8,4	129	54	2,3
Mean	24	29	5,3	70	15	0,9
Std. Deviation	11	23	1,4	26	17	0,62
Std. Error of Mean	2,9	7,4	0,44	6,8	4,3	0,16

**STEATOSIS GRADE 1**	**SEMA3A**	**SEMA3C**	**SEMA4A**	**SEMA4D**	**SEMA5A**	**SEMA7A**
Minimum	6,9	11	4,3	13	0,67	0,03
25% Percentile	16	13	4,7	54	4	0,38
Median	23	16	5,4	67	18	1,2
75% Percentile	34	28	7,8	77	56	1,9
Maximum	54	40	21	129	72	4
Mean	25	20	7,1	69	27	1,2
Std. Deviation	12	9,2	4,5	28	27	1
Std. Error of Mean	2,5	2,5	1,2	6	5,7	0,22

SUPPLEMENTARY TABLE S3.
Serum concentrations of semaphorins according to fibrosis stage in patients with non-alcoholic fatty liver disease

**FIBROSIS STAGE 3 and 4**	**SEMA3A**	**SEMA3C**	**SEMA4A**	**SEMA4D**	**SEMA5A**	**SEMA7A**
Minimum	6,9	64	6	13	0,24	0,1
25% Percentile	12	68	6,1	65	2,2	0,47
Median	15	88	6,4	84	5,5	0,82
75% Percentile	20	99	9,8	114	10	1,1
Maximum	28	99	11	135	33	2,3
Mean	15	85	7,4	85	7,5	0,86
Std. Deviation	5,5	17	2,3	33	8,3	0,54
Std. Error of Mean	1,5	8,3	1,2	8,9	2,2	0,15

**FIBROSIS STAGE 2**	**SEMA3A**	**SEMA3C**	**SEMA4A**	**SEMA4D**	**SEMA5A**	**SEMA7A**
Minimum	7,8	21	4	34	0,13	0,04
25% Percentile	11	21	4,4	52	3,4	0,99
Median	16	26	6,7	67	6,6	1,1
75% Percentile	21	42	10	98	31	1,7
Maximum	33	53	13	129	56	2,6
Mean	16	30	7,1	76	15	1,2
Std. Deviation	6,7	13	3,6	29	17	0,77
Std. Error of Mean	1,7	5,9	1,6	7,4	4,4	0,2

**FIBROSIS STAGE 1**	**SEMA3A**	**SEMA3C**	**SEMA4A**	**SEMA4D**	**SEMA5A**	**SEMA7A**
Minimum	14	9,2	3,5	29	0,22	0,03
25% Percentile	22	13	4,5	43	1,2	0,44
Median	24	16	5	58	8,7	0,77
75% Percentile	41	24	8,5	69	23	1,5
Maximum	68	37	17	129	54	4,7
Mean	30	19	6,8	60	14	1,2
Std. Deviation	13	9	4	23	16	1,1
Std. Error of Mean	2,6	2,2	0,96	4,5	3	0,22

**NO FIBROSIS**	**SEMA3A**	**SEMA3C**	**SEMA4A**	**SEMA4D**	**SEMA5A**	**SEMA7A**
Minimum	10	5,4	3,5	35	0,37	0,03
25% Percentile	22	13	4,7	62	2,9	0,48
Median	23	15	5,6	67	14	0,83
75% Percentile	26	30	7,6	75	56	1,3
Maximum	35	88	21	114	72	2,4
Mean	24	23	6,8	70	24	0,92
Std. Deviation	5,7	16	3,6	19	25	0,61
Std. Error of Mean	0,91	2,6	0,57	3,1	4,1	0,098
